# The Clinical Effectiveness and Cost-Effectiveness of Screening for Age-Related Macular Degeneration in Japan: A Markov Modeling Study

**DOI:** 10.1371/journal.pone.0133628

**Published:** 2015-07-27

**Authors:** Hiroshi Tamura, Rei Goto, Yoko Akune, Yoshimune Hiratsuka, Shusuke Hiragi, Masakazu Yamada

**Affiliations:** 1 Division of Medical Information Technology & Administration Planning, Kyoto University Hospital, 54 Shogoin-Kawahara-cho, Sakyo-ku, Kyoto 606–8507, Japan; 2 Department of Ophthalmology & Visual Sciences, Kyoto University Graduate School of Medicine, 54 Shogoin-Kawahara-cho, Sakyo-ku, Kyoto 606–8507, Japan; 3 Hakubi Center of Advanced Research, Kyoto University, Yoshida, Sakyo, Kyoto 606–8501, Japan; 4 Graduate School of Economics, Kyoto University, Yoshida, Sakyo, Kyoto 606–8501, Japan; 5 National Institute of Sensory Organs, National Hospital Organization Tokyo Medical Center, Tokyo, Japan; 6 Department of Health and Welfare Services, National Institute of Public Health, Wako, Japan; 7 Department of Ophthalmology, Kyorin University School of Medicine, Tokyo, Japan; Tufts University, UNITED STATES

## Abstract

**Objective:**

To investigate the cost-effectiveness of screening and subsequent intervention for age-related macular degeneration (AMD) in Japan.

**Methods:**

The clinical effectiveness and cost-effectiveness of screening and subsequent intervention for AMD were assessed using a Markov model. The Markov model simulation began at the age of 40 years and concluded at the age of 90 years. The first-eye and second-eye combined model assumed an annual state-transition probability, development of prodromal symptoms, choroidal neovascularization (CNV), and reduction in visual acuity. Anti–vascular-endothelial-growth-factor (anti-VEGF) intravitreal injection therapy and photodynamic therapy (PDT) were performed to treat CNV. Intake of supplements was recommended to patients who had prodromal symptoms and unilateral AMD. Data on prevalence, morbidity, transition probability, utility value of each AMD patient, and treatment costs were obtained from published clinical reports.

**Results:**

In the base-case analysis, screening for AMD every 5 years, beginning at the age of 50 years, showed a decrease of 41% in the total number of blind patients. The screening program reduced the incidence of blindness more than did the additional intake of supplements. However, the incremental cost-effectiveness ratio (ICER) of screening versus no screening was 27,486,352 Japanese yen (JPY), or 259,942 US dollars (USD) per quality-adjusted life year (QALY). In the sensitivity analysis, prodromal symptom-related factors for AMD had great impacts on the cost-effectiveness of screening. The lowest ICER obtained from the best scenario was 4,913,717 JPY (46,470 USD) per QALY, which was approximately equal to the willingness to pay in Japan.

**Conclusions:**

Ophthalmologic screening for AMD in adults is highly effective in reducing the number of patients with blindness but not cost-effective as demonstrated by a Markov model based on clinical data from Japan.

## Introduction

Age-related macular degeneration (AMD) is the leading cause of blindness in developed countries.[1–6] In Japan, AMD ranks fourth among the causes of visual impairment, and about 700,000 patients suffer from the disease.[7, 8] Although no treatment for AMD has been available until recently, AMD is now commonly treated with anti–vascular-endothelial-growth-factor (anti-VEGF) intravitreal injection therapy and photodynamic therapy (PDT).[9] AMD frequently develops in only one eye of an elderly person; binocular involvement of AMD is minor[10, 11] because of slow involvement of the other eye.[12–14] Patients may be unaware of their vision changes in daily life when only one of the two eyes is affected, because the impairment of quality of life (QOL) may be limited. Consequently, it may be difficult to detect early-stage AMD, particularly monocular AMD, and subsequent adequate treatment of AMD may be delayed. In addition, treatment for AMD has economic costs to the patients and to society, and the injections require frequent visits to medical institutions over a long period. Thus, conclusions on the reported cost-effectiveness of therapies for AMD are not consistent.[15–17]

Early detection of AMD, however, increases the likelihood of better visual acuity results with the improvement of treatment[18] and of detecting imaging technology[19]. Intake of supplements is also reported to delay the onset of AMD.[20–22] For these reasons, early detection of AMD is considered important. This article addresses the cost-effectiveness of screening for AMD in adults for the critical early detection.

Previous studies on the cost-effectiveness of AMD treatment have identified three important factors influencing their results:[15]

1. the model selected for verification (a second-eye model, a first-eye model, or a combination first-eye and second-eye model);[23]
2. the time horizon;[24]
3. the scope of costs (e.g., whether long-term nursing care costs or indirect costs are included).[17]

Many studies assessing the cost-effectiveness of AMD therapies have used the second-eye model proposed by Brown.[25] Accordingly, their assumption is that therapy is not initiated until the disease develops in the second eye (the previously unaffected eye). This model does not always represent the actual treatment procedure. However, a smaller number of studies have employed the first-eye and second-eye combined model,[15] which more accurately reflects actual treatment procedure. Since screening aims at early disease detection and early intervention based on such early detection, consideration must be given to the first eye.

The Markov model describes repetitive events that occur over a long period. It has been widely used in studies on cost-effectiveness.[26, 27] In fact, the Markov model has been widely used to evaluate the cost-effectiveness of strategies to treat or prevent various ophthalmologic diseases and is therefore the gold standard method for such economic evaluation.[28, 29] The aim of this study was to investigate the clinical effectiveness (reduction in the number of patients with blindness) and the cost-effectiveness (from a value-for-money perspective) of screening and subsequent treatment of AMD in Japan. Specifically, our aim was to use a Markov model to evaluate the reduction in the incidence of blindness and the incremental cost-effectiveness of screening for AMD and subsequent treatment of AMD.

## Participants and Methods

**Fig 1:**
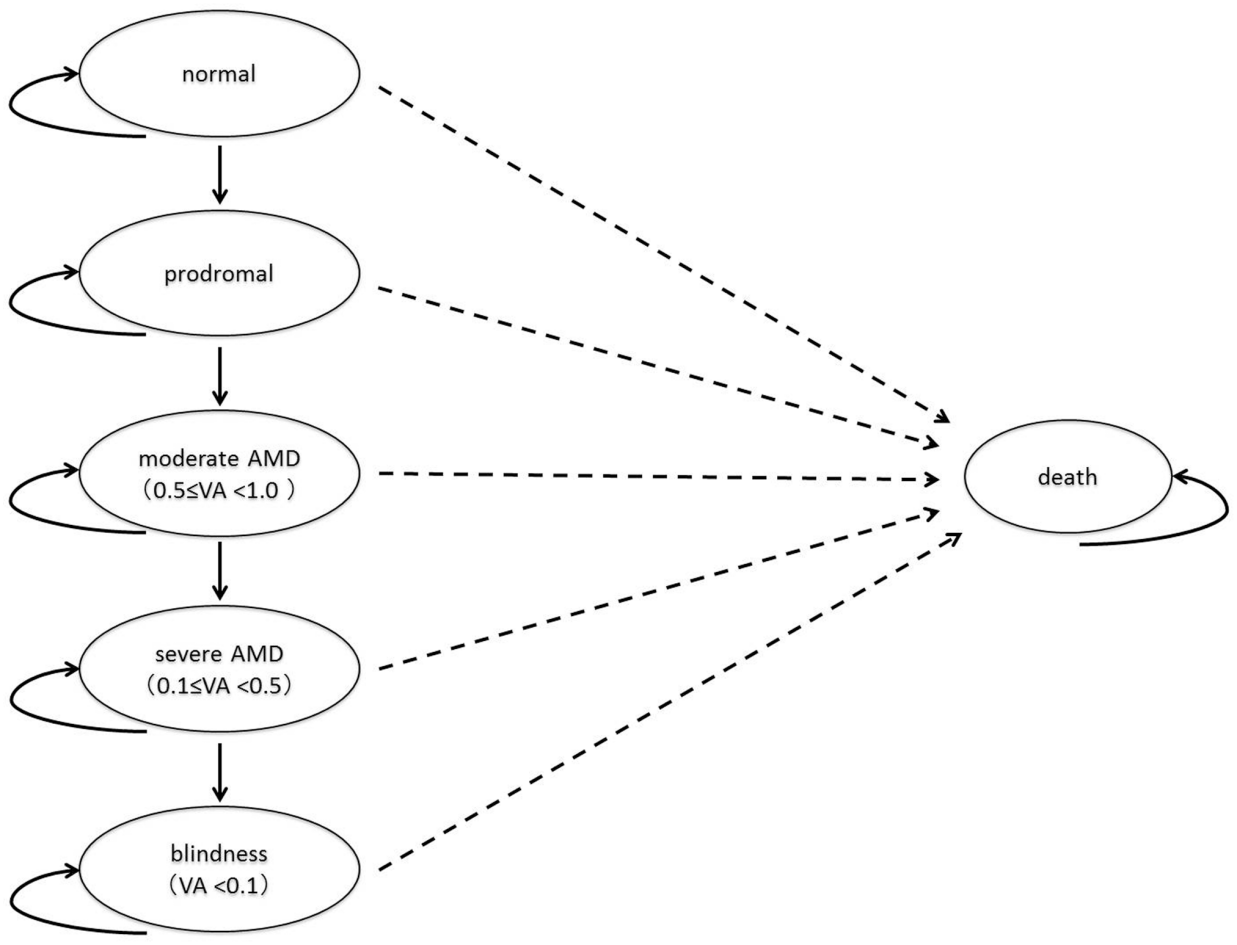
Basic Concepts of Markov Models for AMD. In the current study, AMD is assumed to develop from a normal eye via prodromal state. AMD is categorized in three stages: moderate AMD, severe AMD, and blindness. VA: best corrected visual acuity.

In this study, a model was created and analyzed by using TreeAge Pro 2009 Suite (Release 1.0) to estimate screening outcomes. The model consisted of a decision tree and a Markov model. The decision tree was used to group adults into those receiving ophthalmologic screening (the screened group) and those not receiving screening (the non-screened group). The strategy of the non-screened group was considered representative of the current actual practice for AMD, which involves visits and treatments. The Markov model of disease progression compared two strategies: a screening strategy and non-screening strategy. The first-eye and second-eye combined model was employed. The steps of disease progression were categorized by the following six Markov states (Fig 1): normal, prodromal, moderate AMD, severe AMD, blindness, and death. The severity of AMD was classified into one of the following three stages based on best corrected visual acuity (VA): moderate (VA: 0.5–0.9), severe (VA: 0.1–0.4), and blindness (VA: < 0.1). The model simulated a hypothetical Japanese cohort of 50,000 persons aged 40 years without any eye disease, and the simulation ran until they reached 90 years of age or died according to age-specific background mortality based on the 2009 Japan abridged life tables. Cumulative costs of screenings and treatments and quality-adjusted life years (QALYs) were calculated per person for the entire simulation period, using an annual discount rate of 3%.

Based on published data of incidence rates, it was assumed that the cohort would develop prodromal symptoms and then moderate AMD. In each cycle, each patient’s disease condition was rated as aggravated, maintained, or improved using transition probabilities. For members of the non-screened group, the disease was detected during a coincidental consultation or consultation due to subjective symptoms. For members of the screened group, the disease was detected during a coincidental consultation, consultation due to subjective symptoms, or screening visit. Coincidental consultations refer to visits to ophthalmologic clinics for treatment of eye diseases unrelated to AMD. Consultations due to subjective symptoms refer to visits to ophthalmologic clinics because of subjective symptoms. In the model, increased severity referred to loss of vision in one or both eyes. Visits for screening began at age 50 years, occurred once every 5 years, and ended at age 90 years (base case).

It was assumed that once AMD was detected it was under medical control, with patients receiving the appropriate treatments; the eyes with prodromal symptoms or AMD were examined periodically, the patients were advised to take supplements, and the patients received treatment with intravitreal injection (of anti-VEGF drug) and/or PDT, depending on the stage of AMD.

### Model parameters

Table 1 shows the parameters used in our model. For these parameters, clinical research data on the Japanese population was obtained to the extent possible; if no applicable Japanese data were available, overseas data were used. For the sensitivity analysis, the range of values for each parameter was set for the 95% confidence interval (CI) or ±50% from the reported baseline value.

**Table 1:** Parameter values used in the Markov model with ranges used for univariate sensitivity analysis.

Model parameters group	Model parameters	Classification	Base-case value	Range for univariate sensitivity analysis	References
Probability of initial state					
	aged 40 years	normal	100%	-	
	Age to terminate model		90 years	-	
	Age to start screening		50 years	40, 50, 60, 70 years	
	Age to end screening		90 years	60, 70, 80, 90 years	
	Interval between screenings		5 years	1–10 years	
**Transition probabilities**					
	Normal → prodromal	age 40 years	0.00%	±50%	
		age 45 years	0.95%	±50%	[30]
		age 55 years	0.13%	±50%	[30]
		age 65 years	0.76%	±50%	[30]
		age 75 years	1.40%	±50%	[30]
	Prodromal → moderate	age 40 years	0.00%	±50%	
		age 50 years	0.50%	±50%	[31]
	Prodromal → moderate AMD (second eye)		2.59%	0.20%–4.98%	[32]
	Moderate AMD → severe AMD		37.49%	±50%	[33]
	Severe AMD → blindness		19.07%	±50%	[33]
**Epidemiology**					
	**AMD type**				
		tAMD	40.8%	35.2%–45.3%	[34]
		PCV	54.7%	48.9%–60.4%	[34]
		RAP	4.5%	-	[34]
	Mortality rate		Census of 2009		
**Rates in screening**					
	**Participation rate for screening**				
		Screening	60.00%	30.00%–100.00%	
		Detailed examination	80.00%	30.00%–100.00%	
		Occasional (irregular) screening	20.00%	10.00%–50.00%	
	Incidence rate of presbyopia		3.00%	1%–5%	[35]
	Prevalence of presbyopia	prodromal/moderate AMD	20.00%	-	
		severe AMD	20.00% (in year 1 of stage change)	20.00% (between years 1 and 3 of stage change)	
	Consultation rate caused by increased severity	Better eye and/or worse eye: blindness	100.00%	-	
	**Sensor**				
		Moderate/severe AMD	5.00%	0.00%–50.00%	
		Blindness	0.00%	-	
	**Detection rate in screening**				
		Sensitivity	80%	60%–100%	
		Specificity	95%	80%–100%	
**Utility**		better eye/worse eye			
	Normal	normal/normal	1.00	-	
		normal/ prodromal	1.00	-	
		normal/moderate	0.92	0.87–0.97	[25]
		normal/severe	0.90	0.83–0.92	[25]
		normal/blindness	0.88	0.81–0.92	[25]
	Prodromal	prodromal/prodromal	0.97	0.92–1.00	[25]
		prodromal/moderate	0.92	0.87–0.97	[25]
		prodromal/severe	0.90	0.83–0.92	[25]
		prodromal/blindness	0.88	0.81–0.92	[25]
	Moderate AMD	moderate/moderate	0.85	0.73–0.96	[36]
		moderate/severe	0.83	0.73–0.85	[36]
		moderate/blindness	0.81	0.71–0.85	[36]
	Severe AMD	severe/severe	0.57	0.46–0.85	[36]
		severe/blindness	0.55	0.46–0.57	[36]
	Blindness	blindness/blindness	0.46	0.35–0.57	[36]
**Cost**					
	Screening		3,000 JPY	±50%	
	Detailed examination		13,900 JPY	±50%	
	Observation		4,420 JPY	±50%	
	Supplements (1 year)		51,360 JPY	±50%	
	Ranibizumab (each time for one eye)		182,035 JPY	±50%	
	PDT (each time for one eye)		363,450 JPY	±50%	
	PDT (each time for both eyes)		540,000 JPY	±50%	
	Endophthalmitis		1,052,750 JPY	±50%	
Discount rate			3.00%	0.00%–5.00%	
**Treatments**					
	**Prodromal (supplements)**				
	Intake rate		50%	0%–100%	
	Rate of continuation of supplement intake each year		90%	50%–100%	
	Rate of suppression of AMD		25%	0%–50%	[21]
	Number of observations per year		4	2–6	
	**Moderate AMD (ranibizumab)**				
	Number of observations per year		12	6–12	
	Number of injections per year	year 1	6	3–12	
		year 1	4	2–8	
		after year 2	2	1–3	
	Effect of treatment	maintain	95%	-	
		moderate AMD → severe AMD	5%	1%–10%	
	**Severe AMD (ranibizumab and PDT)**				
	Number of observations per year		12	6–12	
	Number of injections per year	year 1	6	3–12	
		year 2	4	2–8	
		after year 2	2	1–3	
	Number of PDT treatments per year	year 1	1	1–6	
		after year 1	0	-	
	Effect of treatment	severe AMD →moderate AMD	40.3%	32.2%–48.5%	[37]
		maintain	54.7%	-	
		severe AMD → blindness	5%	1%–10%	
		moderate AMD → severe AMD after year 1	22%	5%–40%	[38]
	Number of observations of each blind patient per year		12	6–12	
	Incidence rate of Endophthalmitis per injection		0.03%	0.01%–0.05%	[39]

Abbreviations: PDT (photodynamic therapy), tAMD (typical neovascular AMD), PCV (polypoidal choroidal vascularization), RAP (retinal angiomatous proliferation).

### Model validation

To validate our model, we compared the reported numerical values (for prevalence of AMD, incidence of unilateral AMD, and rate of blindness) with simulated values, which are described later, for the non-screened group. To simulate the future number of AMD patients and to validate the model, the number of AMD patients after controlling ages was calculated by using as a starting point the current population, by age, in each Markov status.

### Ophthalmologic screening program

It was assumed that ophthalmologists used fundus photographs in making their diagnoses.

### Morbidity rate

AMD was assumed to develop in eyes with prodromal symptoms. Probabilities of the onset of prodromal symptoms and AMD (one eye) in the Japanese population were obtained from published reports.[30, 31, 40] When AMD developed in only one eye, the probability of AMD occurring in the unaffected eye was quite high. The probability of AMD occurring in the second eye was obtained from the literature and used in cases of prodromal stage disease being detected in the second eye.[12–14]

### Participation rate for screening

Because there is currently no concrete and consistent ophthalmologic screening program for adults, data on the participation rates for screening and detailed examination are limited. Arbitrary numbers for these parameters were set in the base-case analysis and sensitivity analysis was performed to evaluate the effect of these parameters on the model.

Although the coincidental consultation may take place in a variety of clinical settings, our model assumed that patients visited ophthalmologic clinics with presbyopia or AMD at specific rate. The prevalence of presbyopia in Japan is reported to be 43.8% for those aged 40 years or older,[35] and our estimate of the annual rate of increase in presbyopia was 3%. We assumed that 20% of those with presbyopia would visit ophthalmologic clinics and be diagnosed with AMD. However, the probability of these assumptions was applicable only for a 1-year period after the onset of presbyopia.

### Dropout from treatment

In the early stages of AMD, patients rarely notice their symptoms, thus their motivation to continue treatment is weak, leading to some drop out of treatment. Our model assumed that patients with prodromal symptoms (therefore at high risk for AMD) would undergo periodic monitoring of symptoms and receive supplement therapy. Dropouts in this group were expected to be particularly high: the rate of withdrawal was set at 50% for the first year of therapy and at 10% for subsequent years. For patients with later stages of the disease, the rate of withdrawal was set at 5%. For patients with unilateral or bilateral blindness, no withdrawal was considered because of their high adherence to medical services.

### Prognosis and therapy

The progression of a patient through the stages of AMD was characterized by transition probabilities obtained from published reports.[33, 37, 41–48] For prodromal symptoms, supplement intake, which reduces the incidence of AMD,[21, 49, 50] was assumed in addition to follow-up observations.

Treatment of AMD depended on its stage and type. AMD is classified into one of three types: typical neovascular AMD (tAMD), polypoidal choroidal vascularization (PCV), and retinal angiomatous proliferation (RAP). In our model, the probability of a patient having each type of AMD was based on previously published rates of AMD onset.[34] It was assumed that the type of AMD affecting the second eye would be the same as that affecting the first eye. The treatment strategies assumed in our model were in accordance with a Japanese clinical guideline.[51] Specifically, intravitreal injection (of ranibizumab) was administered to those with moderately severe conditions irrespective of AMD type. For those who did not drop out of a series of treatments, the number of injections per year was set at 6 for the first year of treatment, 4 for the second year, and 2 for the third year and each subsequent year, based on actual world data.[52] It was assumed that the treatment would maintain the visual acuity in 95% of patients but that disease severity would increase in the remaining 5%. Beginning in the second year of treatment, the annual transition probability—the probability of developing a more severe stage of the disease—was assumed to be 22%.[38]

**Fig 2:**
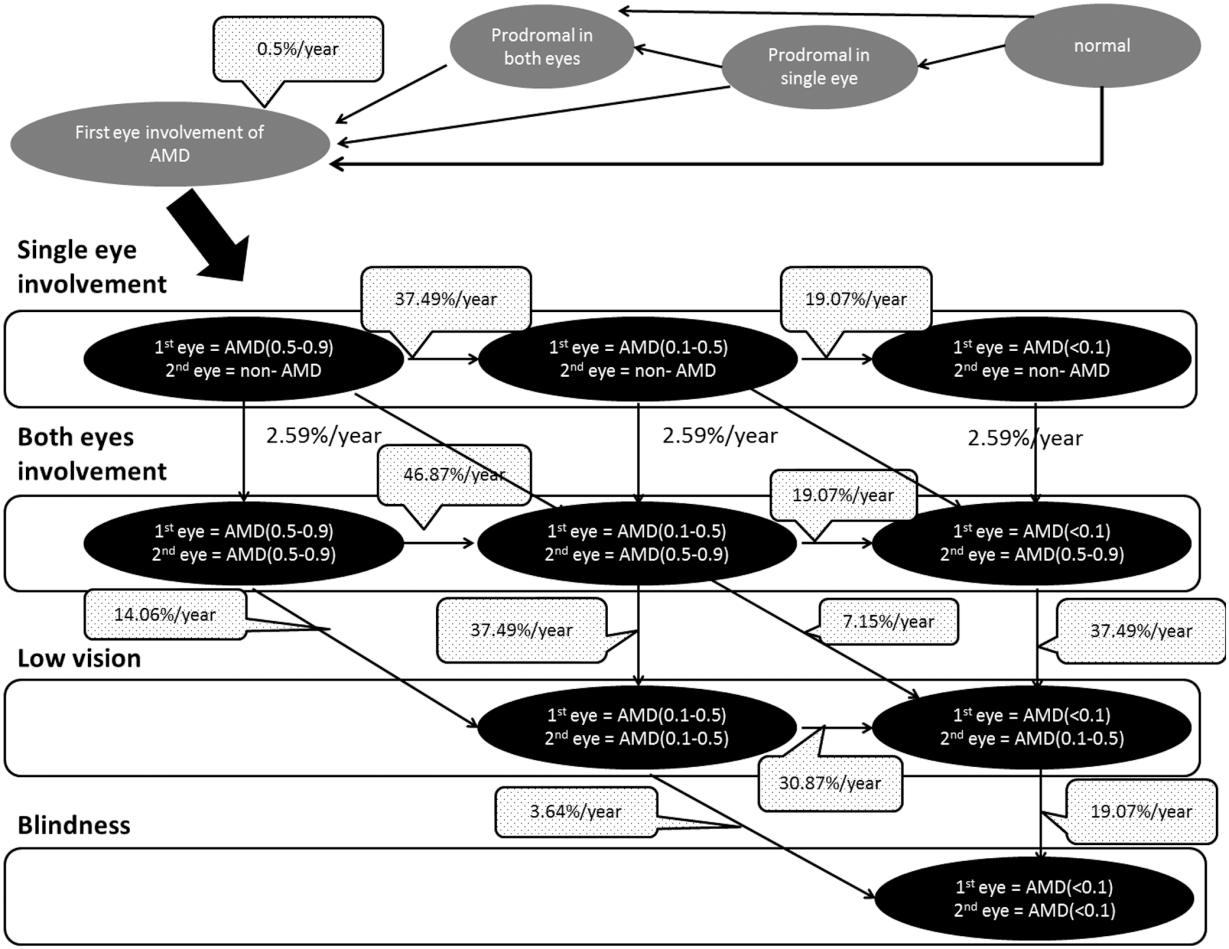
Detailed Markov model structure showing transitions in health status of both first eye and second eye without treatment. Transition probabilities were derived from previous reports, including the Hisayama Study,[30] MARINA,[33] FOCUS[47] and PIER[48].

**Fig 3:**
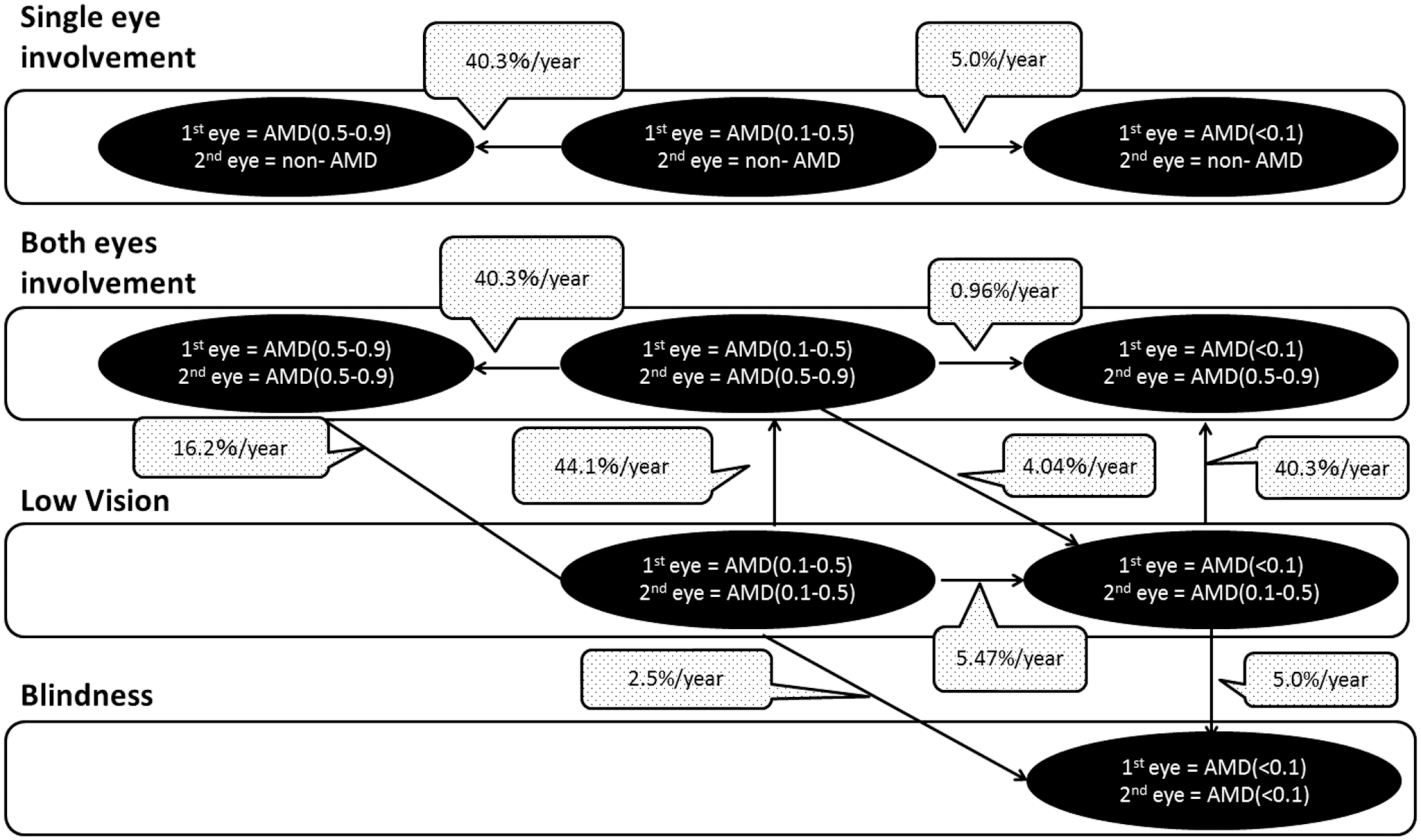
Detailed Markov model structure showing transitions in health status of both first eye and second eye after treatment. Transition probabilities were derived from previous reports, mainly ANCHOR.[37]

When a patient’s disease condition was severe, a patient with tAMD was assumed to receive only intravitreal injection, and a patient with PCV or RAP was assumed to undergo both intravitreal injection and PDT. The number of injections administered to a patient with severe condition was assumed the same as that administered to a patient with moderate condition. PDT was performed only once, in the first year of treatment, and then never again. Since PDT does not substantially contribute to improvement of visual acuity but rather aims primarily at maintaining current visual acuity, the therapeutic prognosis of intravitreal injection (of ranibizumab) was evaluated using outcomes obtained from published reports.[37] The probability of a patient transitioning from severe AMD to moderate AMD in the first year of treatment was set at 40.3%, and from severe AMD to blindness at 5%. During and after the second year of treatment, it was assumed that the disease was severe in 95% of patients. When blind, patients were assumed to receive no treatment but to be under observation. Figs 2 and 3 show the transition in patient health status in the first-eye and second-eye combined model used in the study.

### Clinical Effectiveness Analysis

We calculated the reduction in the rate of blindness and the future number of AMD patients by comparing the base-case analysis values shown in Table 1 to the simulated numbers for each screening strategy.

### Costs

Our model considered only direct medical costs. Specialists estimated the costs of screening, detailed examination required to make a definite diagnosis of AMD, tests and examinations, and various types of treatments, based on reimbursement rates defined in fees schedules for Japanese social health insurance. All costs were in Japanese yen (JPY) and were converted into US dollars (USD) (2014) using Federal Reserve historical foreign exchange rates (1 US$ = ¥ 105.74), accessed on the following web site: https://www.federalreserve.gov/releases/g5a/current/.

### Utility value

The utility value for each AMD patient, or the patient’s preference-based QOL, was set at 1 for a healthy member of the population and at 0.97 for a patient having prodromal symptoms in both eyes. Utility data were obtained using a time trade-off (TTO) method for measuring visual-acuity–specific utility in patients with AMD. However, since the utility value may be somewhat reduced when visual acuity differs between the better-seeing and worse-seeing eye, as mentioned in previous reports,[53, 54] utility value was reduced for the worse-seeing eye to allow the worse-seeing eye to affect utility value, based on those previous reports.[54] The reasonability of the utility value assumptions was also assessed by incorporating ranges in the sensitivity analysis. Table 1 shows the utility value assumed for each stage of AMD.

### Cost-utility analysis

We calculated an incremental cost-effectiveness ratio (ICER) using the base-case analysis values shown in Table 1, to enable comparisons of cost versus utility value for each screening strategy. ICER was calculated using the following formula:

ICER= Incremental Cost / QALY Gained

### Screening program

To determine the optimal screening program, the age at which to start screening, the age at which to end screening, and the interval between screenings were each varied within their respective ranges shown in Table 1, and the resulting ICER was calculated.

### Sensitivity analysis

One-way sensitivity analysis (one-way SA) and probabilistic sensitivity analysis (PSA) based on 10,000 Monte Carlo simulations were performed to assess the influence of each parameter on the base-case results.

### Ethics Statement

All investigations in the current study adhered to the tenets of the Declaration of Helsinki.

## Results

### Model validation

We tested the model’s external validity by comparing the model’s simulated values for the non-screened group (prevalence of AMD in persons 40 years or older, incidence of unilateral AMD, and incidence of blindness due to AMD in persons 40 years or older) to reported values. The prevalence of AMD in the simulated cohort was 1.31%, which was comparable to the 1.30% reported in the Hisayama study.[30, 31] The model’s unilateral AMD incidence of 84.1% was comparable to the 79.3% reported in the Nagahama study.[11] The cumulative incidence of blindness due to AMD was 0.026% in the simulated cohort, which was comparable to the 0.019% estimate reported by the Japan Ophthalmologists Association in 2007.[55] The model was validated, shown to represent adequately, but not perfectly or exactly, the current epidemiologic status.

### Clinical effectiveness analysis

**Fig 4:**
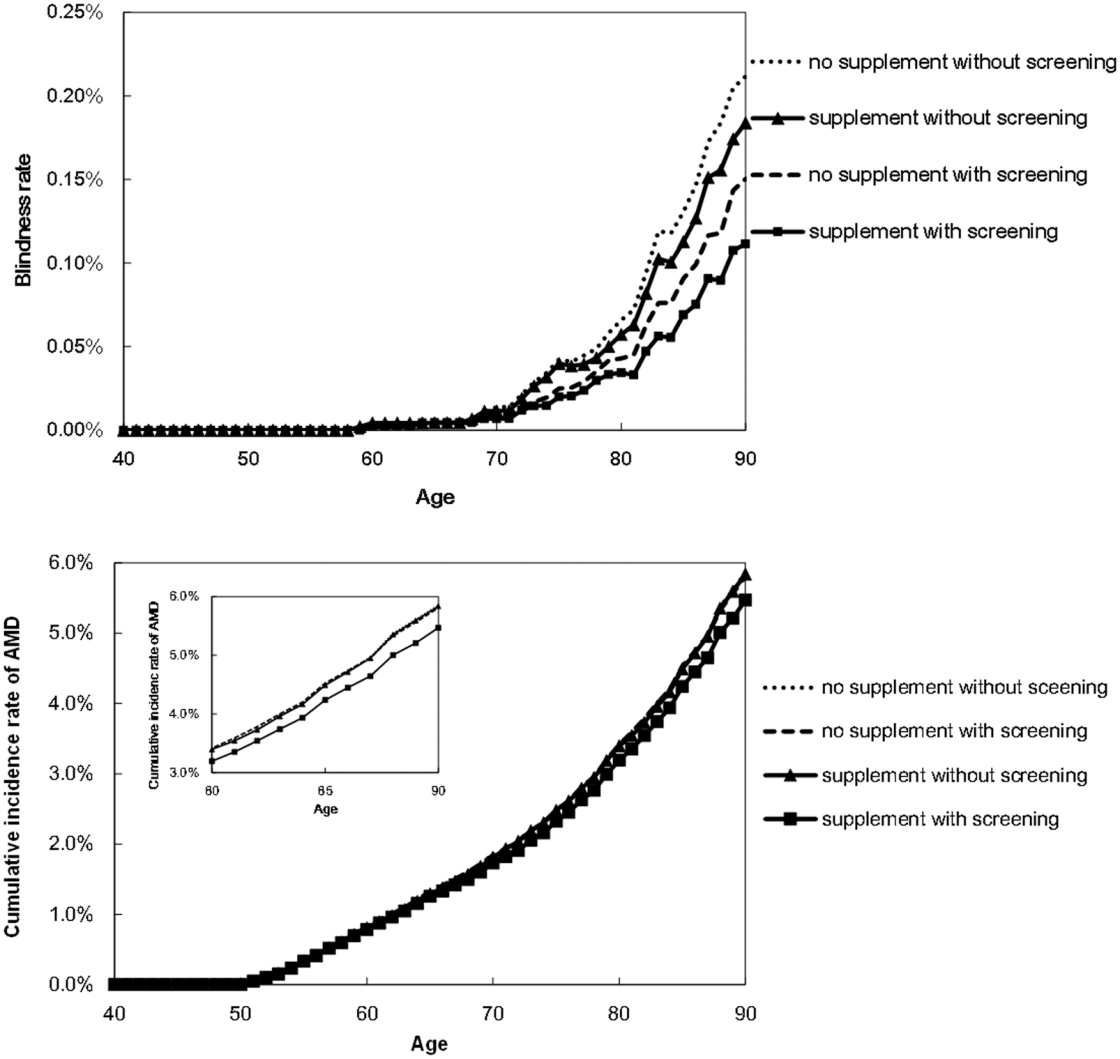
Top: The reduction rate of blindness from age-related macular degeneration, by age. Bottom: The reduction in cumulative incidence of age-related macular degeneration, by age.

Table 2 shows the results of clinical effectiveness in the base case. Screening interventions reduced the rate of blindness by 41% after controlling for age (the crude reduction rate calculated by the Markov model was 37%). AMD incident rate was reduced 5% by supplement intake subsequent to prodromal detection from screening. Fig 4 shows the reduction in rate of blindness and AMD by age. Reduction effect of blindness was more apparent and occurred earlier than did a reduction in the number of AMD patients, and the screening program reduced blindness rate by more than did the additional intake of supplements. The rate of blindness decreased more between no supplements without screening and no supplements with screening than it did between no supplements with screening and supplements with screening (Fig 4, top).

**Table 2:** Base-case analysis in a simulated population.

	Screened	Non-screened
***Clinical effectiveness***		
Number of people at prodromal state	13,095,310	13,049,875
Number of people with AMD	870,956	916,390
Incidence rate of AMD (%)	1.25	1.31
Proportion of patients with AMD in only one eye (%)	86.1	84.1
Number of people with blindness by AMD	7,755	13,142
Blindness rate by AMD per population (%)	0.011	0.019
***Cost-effectiveness***		
Cost (JPY/population)	103,575	22,427
Incremental cost vs. without screening	81,148	-
Utility (QALY)	23.5630	23.5601
Incremental utility vs. without screening	0.0030	-
ICER vs. without screening (JPY/QALY)	27,486,352	-

### Cost-effectiveness analysis

Table 2 also shows the results of cost-effectiveness in the base case. Comparison of the screened group with the non-screened group revealed an incremental cost of ¥81,148, incremental utility of 0.00295, and ICER of 27,486,352 JPY, or 259,943 USD per QALY. The willingness to pay (WTP) in Japan, representing the threshold value of ICER for cost-effectiveness, is considered to be 5,000,000 JPY/QALY, or 47,286 USD/QALY.[56] These results indicate that ophthalmologic screening for AMD is not cost-effective.

### Efficient screening program

The age at which to start screening, age at which to end screening, and interval between screenings were each varied within their respective ranges (Table 1), and an ICER was calculated for the screening group and for the non-screening group. For the screening group, the minimum ICER was 15,003,591 JPY/QALY, in the model for which screening starts at age 50, occurs at 6-year intervals, and ends at age 60 (actually ends at age 56 so that no screening would occur at age 62), and the maximum ICER was 58,593,956 JPY/QALY, in the model for which screening starts at age 60, occurs at 7-year intervals, and ends at age 90 (actually ends at age 88 so that no screening would occur at age 95). In USD, the ICER range is between 141,891 USD/QALY and 554,132 USD/QALY. For all screening programs, both the incremental costs and the incremental utility values of screening versus no screening were positive numbers. However, none of the screening programs had an ICER less than 5,000,000 JPY, Japan’s threshold for cost-effectiveness.

### Sensitivity analysis

**Fig 5:**
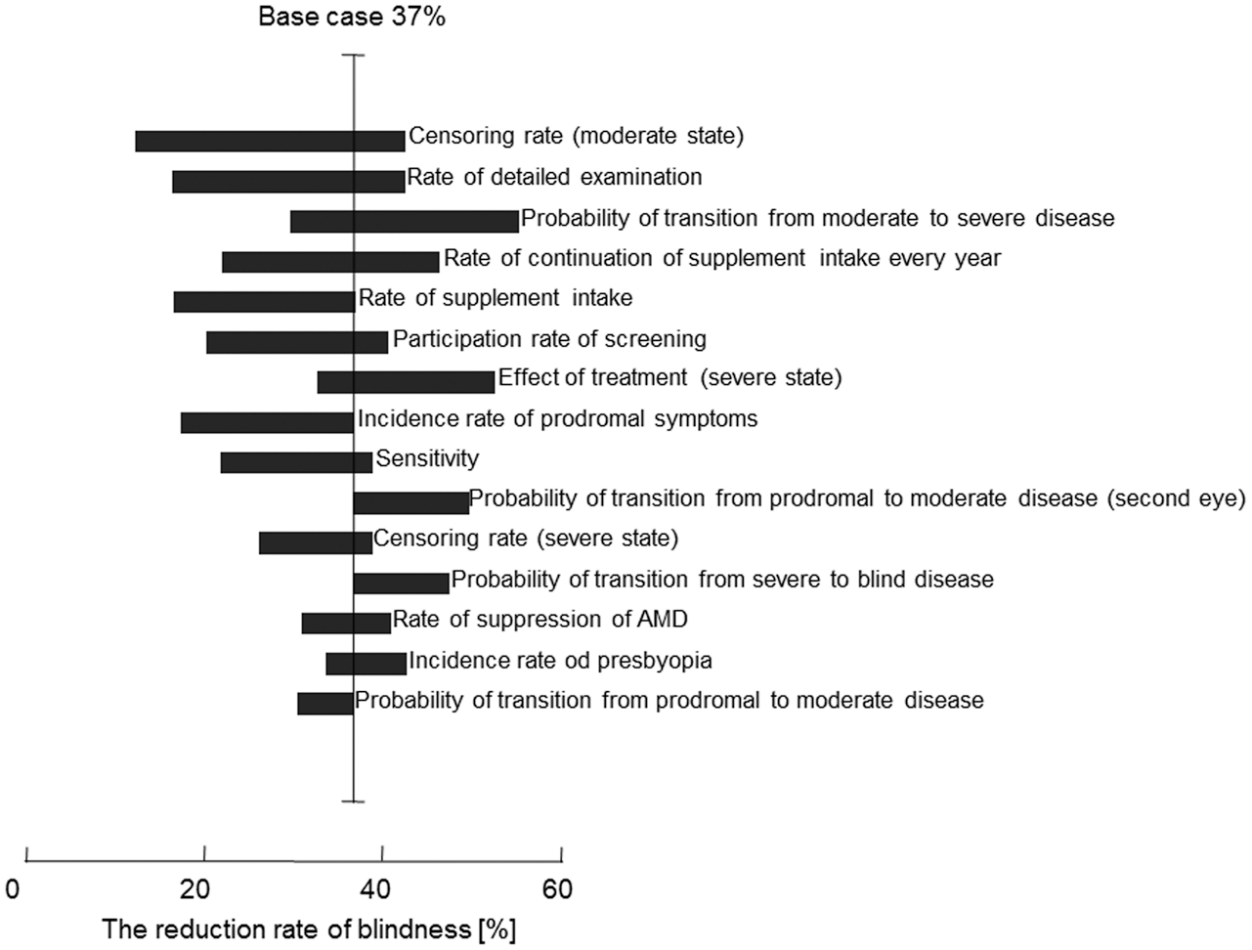
Tornado diagram showing one-way sensitivity analysis targeting reduction of blindness rate as the outcome. The 15 most effective parameters of the 52 total parameters are shown.

Fig 5 is a tornado diagram showing the blindness reduction rate ranges for the 15 most effective parameters among the 52 total parameters. The reduction rates of blindness were calculated for the screened group as compared to the non-screened group. The dropout rate from the recursive treatment for AMD, denoted by “sensor (moderate/severe),” had the greatest effect in our model, resulting in blindness reduction ranging from 12.5% (+50%) to 42.6% (0%) in the sensitivity analysis. Several factors that lacked sufficient evidence included “detailed examination,” “rate of continuation of supplement intake every year,” “rate of supplement intake.” Furthermore, screening participation rate might weaken other factors’ effects on reduction of blindness rate.

**Fig 6:**
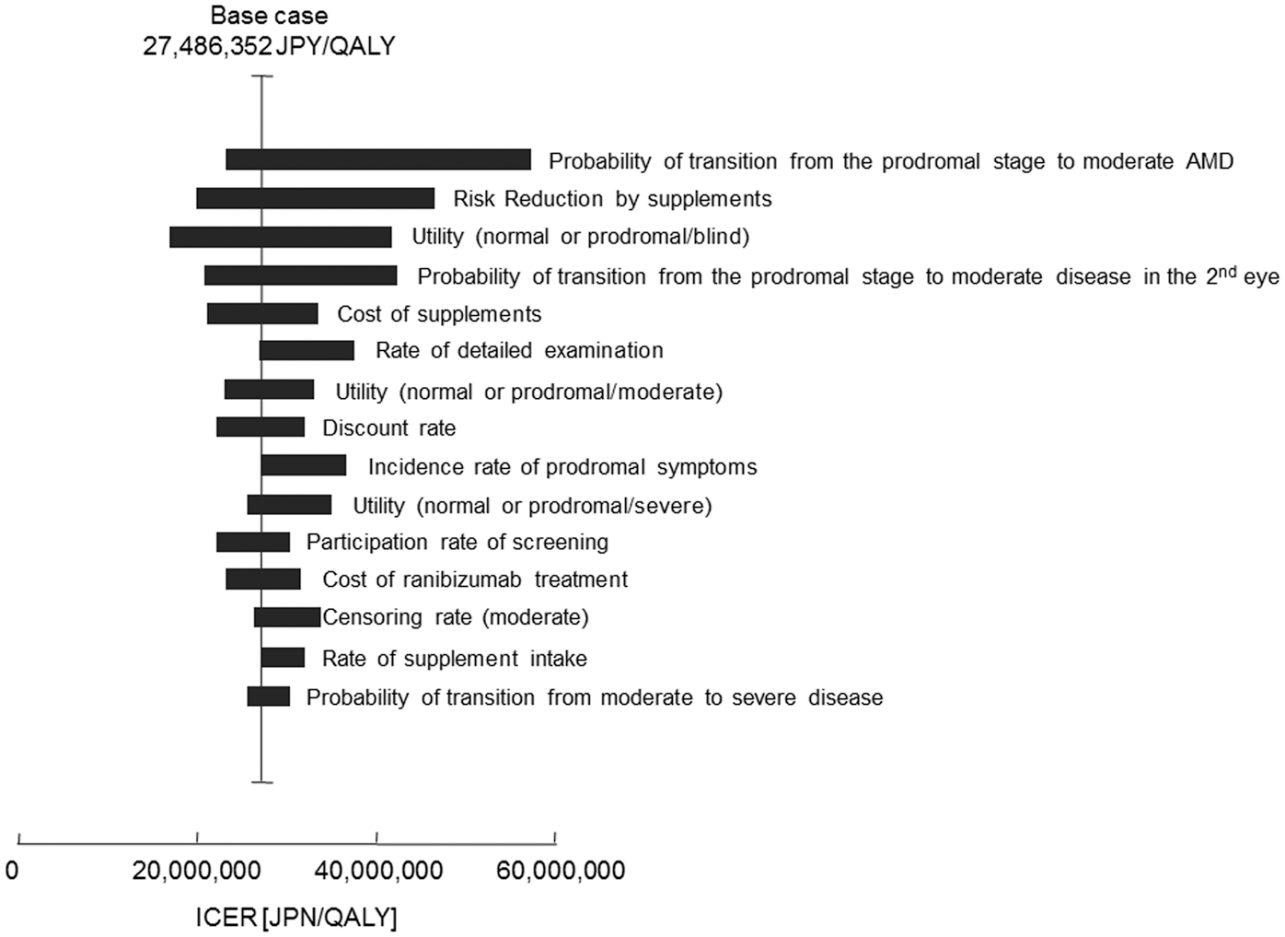
Tornado diagram showing one-way sensitivity analysis targeting incremental cost-effectiveness ratio (ICER) as the outcome. The 15 most effective parameters of the total 52 parameters are shown.

Fig 6 is a tornado diagram showing the ICER ranges for the 15 most effective parameters among the 52 total parameters. ICERs were calculated for the screened group as compared to the non-screened group. The probability of transition from the prodromal stage to moderate AMD had the greatest effect in our model, resulting in ICER ranging from 23,552,326 JPY/QALY (222,738 USD/QALY) (+50%) to 57,321,478 JPY/QALY (542,098 USD/QALY) (-50%) in the sensitivity analysis. Other prodromal symptom-related factors for AMD, including “risk reduction by supplements,” “probability of transition from the prodromal stage to moderate disease in the second eye,” and “cost of supplements” also had major effects on cost-effectiveness. When it was assumed that the drug was switched from ranibizumab to bevacizumab (but with the same therapeutic effect), the ICER decreased to 19,906,587 JPY/QALY (188,260 USD/QALY). In a best-case scenario for cost-effectiveness, assuming that a patient in the prodromal stage of AMD was followed up once annually, supplements cost 0 JPY, and bevacizumab was used, the ICER decreased to 4,913,717 JPY/QALY (46,470 USD/QALY), approximately equal to the WTP in Japan, while the prophylactic effect against future blindness was maintained at the same level.

### Probabilistic sensitivity analysis

**Fig 7:**
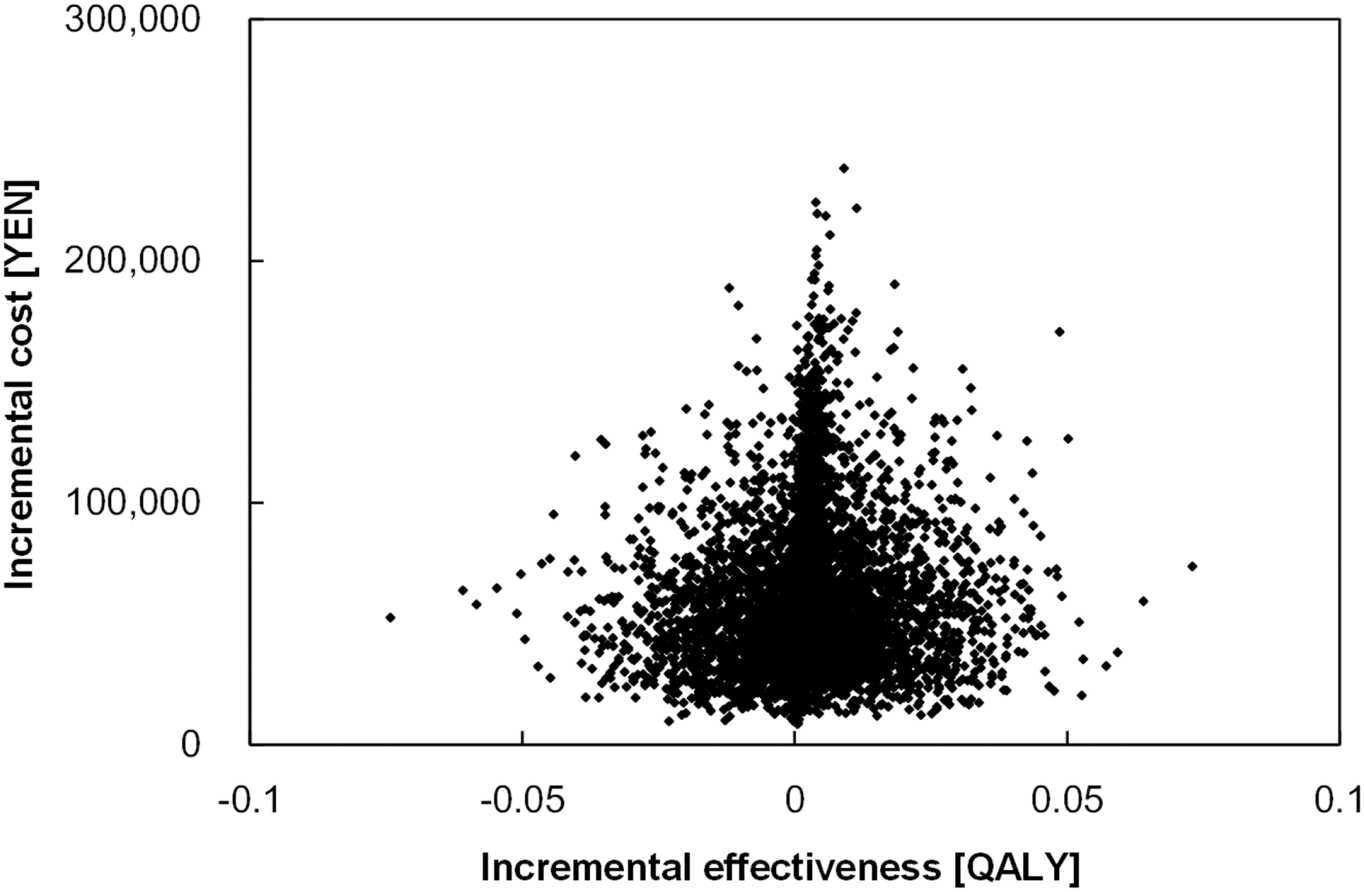
Probabilistic sensitivity analysis of cost-effectiveness analysis for the screening program for age-related macular degeneration.

In Fig 7, the results of PSA are presented graphically. The 10.4% of simulation results located in quadrant 1 are below the WTP threshold of 5,000,000 JPY. The screening program was more effective than the no-screening option in 82.8% of the results, although screening was dominated by no-screening in the remaining 17.2% of the results.

## Discussion

The screening program reduced both the rate of blindness caused by AMD and the cumulative incidence rate of AMD. However, the decrease in the rate of AMD-caused blindness was more apparent and occurred earlier than did the decrease in the incidence of AMD. This comparative success might derive from current medical intervention’s ability to only ameliorate the symptoms or delay the progression of the disease rather than eliminate the disease itself. The screening program evaluated in this study reduced by 41% the cumulative number of patients to suffer blindness from AMD. The reduction is as compared to the non-screening population and assumes current medical intervention with intravitreal injection of inhibitors of vascular endothelial growth factor (VEGF). Without anti-VEGF drug therapy, the reduction would be much greater, because, since the introduction of intravitreally injected anti-VEGF in 2006, the incidence of legal blindness from AMD has already fallen to half the previous baseline incidence rate.[57] The screening program reduced the blindness rate by more than did additional intake of supplements. This comparative findings is confirmed by the rate of blindness decreasing more between no supplement without screening and no supplement with screening than it did between no supplement without screening and supplement without screening (Fig 4, bottom). The screening program’s greater effectiveness than supplement intake has not been well discussed until now. This finding should be confirmed in future discussion of early detection approaches.

Previous studies on the cost-effectiveness of ophthalmologic treatments have frequently used the second-eye model.[17, 23, 43, 58–60]. However, the following assumptions of the second-eye model are not necessarily adequate to the study of cost-effectiveness of the AMD screening program:

1. unilateral AMD assumed not to be treated, whereas in reality, many patients with unilateral AMD (about 80% of patients with AMD suffer from unilateral disease[11]) are treated;
2. the worse eye in bilateral AMD assumed not to be treated, whereas in actual clinical settings, both of the affected eyes are frequently treated; and
3. treatment of the worse eye assumed to be effective but not improving patients’ QOL, whereas in reality, many patients are at least somewhat satisfied with improvement in their worse eyes. Recent studies have reported the worse-seeing eye to have a stronger influence on vision-related QOL than is generally assumed.[54, 61]

Therefore, a first-eye and second-eye combined model, which has been used infrequently,[23, 24] was used in this study.

Some published studies included non-medical costs (such as the cost of long-term care) in the direct costs to be evaluated,[23, 37, 58, 62] while Hurley, et al. indicated that cost-effectiveness is greater when indirect long-term care costs are included.[17] Whether or not indirect long-term care costs should be included in our present analysis was an important issue. We decided not to include these costs in our study, because the costs depend on how the screening program is conducted and where the targeted population is located. If we were to include these costs, the results would be even more cost-ineffective—costs would be higher. Furthermore, the sensitivity analysis showed that the cost of screening had little effect on cost-effectiveness.

The base-case analysis, shown in Table 2, demonstrated that the screening program evaluated in this study reduced the cumulative number of patients with blindness by 41%. Screening for AMD, however, was not cost-effective, because the ICER was above the threshold WTP estimated for Japan. A major factor contributing to this cost-ineffectiveness finding may be the unilaterality of the disease. In our model, patients with unilateral AMD at age 90 accounted for 84.8% of all AMD patients in the screened group and 82.7% of all AMD patients in the non-screened group. The utility value, or QALY, of these patients would not be improved even by expensive treatments such as anti-VEGF drugs for worse eyes, because QALY is defined by the condition of the better eye (the unaffected eye).[25] In this study, we incorporated the real-life situation, allowing the worse eye to affect utility value, by reducing utility values when visual acuity differed between the better and the worse eye. In the future, cost-utility analysis using utility values based on actual TTO methods should be incorporated when the eyes of an AMD patient differ in visual acuity. Further study is needed to strengthen the opinion we have derived from this study’s results.

In this study, we relied on data regarding treatment mainly from reports of ideal randomized controlled trials with two-year follow-up periods, because they were the only ones available. Recently, outcomes from actual clinics with relatively long follow-up periods have been reported.[63] Using the newly available data may worsen the cost-effectiveness result, because visual acuity was reported to decrease after the first two years and to be even worse than baseline after four years.

One-way sensitivity analysis revealed that the probability of a patient’s transition to AMD, the prodromal symptom-related factors (including the preventive effect of supplement intake and the cost of supplements), and utility values had the greatest effects on the model, but those effects were not sufficiently large to make screening intervention cost-effective. In addition, discount rates also affected the cost-effectiveness of screening, because there is a long interval of time between screening and the confirmation of its effectiveness.[24] Accordingly, it is desirable to use a common discount rate across studies that compare similar types of medical procedures; as a result, we employed 3% in this study. With regard to utility values, when the fellow eyes of normal or prodromal eyes is affected by AMD (when better-seeing eye is normal or prodromal, while worse-seeing eye is blind, sever AMD or moderate AMD), they impacted the results. Although previous reports have paid little attention to the phenomenon, our results showed that such difference in utility values when the two eyes are in different stages should be considered. Further research on such differing utility values when the two eyes are in different stages should be incorporated in studies of the cost-effectiveness of treatment for one-eye–dominant disease.

This study suggests that factors regarding treatment and progression in early stages of AMD are important, influencing the analysis of cost-effectiveness. This may be because, as shown in the Table 2, screening remarkably increases detection of the prodromal stage of AMD. The cost of intravitreal injection of ranibizumab also had an impact. When bevacizumab was substituted for ranibizumab and both drugs were assumed to have the same therapeutic effects, the ICER decreased only to 19,906,587 JPY/QALY (188,260 USD/QALY). Thus, screening only for AMD is not cost-effective.

When screening for AMD, however, other vision-threatening ocular diseases, including glaucoma and diabetic retinopathy, can be detected. A comprehensive approach to ocular disease screening, in which AMD is one of several diseases that could be detected, may yield better cost-effectiveness, because single screenings for other ocular diseases are reported to be cost-effective.[64] On the other hand, sensitivity analysis revealed that screening cost had only a limited effect on ICER. In other words, even though the incremental cost of medical examination in the screening is low, particularly when no additional screening procedure is conducted to detect AMD, the cost-effectiveness of screening will not be much improved. The cost-effectiveness of both AMD screening included in a comprehensive ocular disease screening and an entirely separate AMD screening is not high.

## Conclusion

We evaluated the clinical effectiveness and cost-effectiveness of ophthalmologic screening for AMD in adults using a Markov model based on clinical data from Japan. In evaluating the AMD screening program from the viewpoint of clinical effectiveness (reduction in the number of patients with blindness) and cost-effectiveness (from a value-for-money perspective), our results indicate that the screening program is highly effective in reducing the number of patients with blindness but not cost-effective. The PSA results confirm that the screening program is beneficial in terms of health gain in most cases but that its cost-effectiveness is low given the present ICER threshold (WTP). Although parameter uncertainty has a great effect on analysis of the results, further epidemiological and prognostic knowledge about AMD would lead to more robust cost-effectiveness research.
